# Diffusion-Weighted MRI for Treatment Response Assessment in Osteoblastic Metastases—A Repeatability Study

**DOI:** 10.3390/cancers15153757

**Published:** 2023-07-25

**Authors:** Maria Eveslage, Philipp Rassek, Arne Riegel, Ziad Maksoud, Jochen Bauer, Dennis Görlich, Benjamin Noto

**Affiliations:** 1 Institute of Biostatistics and Clinical Research, University of Münster, 48149 Münster, Germany; 2 Department of Nuclear Medicine, University Hospital Münster, 48149 Münster, Germany; 3 Clinic for Radiology, University Hospital Münster, 48149 Münster, Germany

**Keywords:** apparent diffusion coefficient, diffusion-weighted imaging, quantitative imaging, bone metastases, prostate cancer, repeatability, test–retest study

## Abstract

**Simple Summary:**

Patients with many advanced cancers develop osteoblastic bone metastases that cannot be assessed by conventional imaging. There is an unmet need for a quantitative imaging technique that can assess the treatment response of osteoblastic metastases to further improve treatment of these patients. This article examines the difference in apparent diffusion coefficient (ADC) values found between viable and nonviable metastases in relation to the variability of repeated measurements as a basis for the potential use of diffusion-weighted MRI (DWI) for treatment response assessment. DWI is based on observing the movement of water molecules, which is often restricted in tumor tissue and is quantified using the ADC. It is shown that viable and nonviable metastases differ significantly in ADC value and that these differences are considerably higher than the variability of repeated measurements. This shows that DWI meets the basic technical requirements for reliable treatment response assessment of osteoblastic metastases.

**Abstract:**

The apparent diffusion coefficient (ADC) is a candidate marker of treatment response in osteoblastic metastases that are not evaluable by morphologic imaging. However, it is unclear whether the ADC meets the basic requirement for reliable treatment response evaluation, namely a low variance of repeated measurements in relation to the differences found between viable and nonviable metastases. The present study addresses this question by analyzing repeated in vivo ADC_median_ measurements of 65 osteoblastic metastases in nine patients, as well as phantom measurements. PSMA-PET served as a surrogate for bone metastasis viability. Measures quantifying repeatability were calculated and differences in mean ADC values according to PSMA-PET status were examined. The relative repeatability coefficient %RC of ADC_median_ measurements was 5.8% and 12.9% for phantom and in vivo measurements, respectively. ADC_median_ values of bone metastases ranged from $595\times 10^{-6}\;{\mathrm{mm}}^{2}/s$ to $2090\times 10^{-6}\;{\mathrm{mm}}^{2}/s$ with an average of 63% higher values in nonviable metastases compared with viable metastases (*p* < 0.001). ADC shows a small repeatability coefficient in relation to the difference in ADC values between viable and nonviable metastases. Therefore, ADC measurements fulfill the technical prerequisite for reliable treatment response evaluation in osteoblastic metastases.

## 1. Introduction

The Response Evaluation Criteria In Solid Tumors (RECIST) guidelines are widely accepted for measuring tumor response in solid tumor patients and are used by regulatory agencies for drug approval [1,2,3]. The guidelines are based on morphologic imaging, measuring the size of target lesions and assessing changes in size over time. However, they have limitations in evaluating bone metastases, which can be osteolytic, osteoblastic, or radio-occult [4,5]. Osteolytic metastases are characterized by bone destruction and are measurable if they have a soft tissue component of at least 1 cm, while osteoblastic metastases, characterized by increased bone formation, are generally defined as not measurable [1]. Bone scintigraphy, a molecular imaging technique, also has limitations in distinguishing treatment response from progression [6,7,8]. One of these limitations is that progression can only be ascertained with delay [8]. This is particularly problematic in prostate cancer and breast cancer, with prostate cancer being the second most diagnosed cancer in men and breast cancer being the most common cancer in women [9,10]. Up to 70% of patients with breast and prostate cancer have evidence of metastatic bone disease in advanced stages. Bone metastases of prostate cancer are almost exclusively osteoblastic and therefore virtually always not measurable within the RECIST framework, while breast cancer often presents with mixed osteolytic and osteoblastic metastases, often rendering these metastases unmeasurable as well [11,12]. The inability of current imaging techniques to recognize treatment response or progression in a timely manner can potentially delay treatment switching when disease burden is still relatively low. Therefore, new imaging techniques are needed to provide a reliable assessment of treatment response in bone metastases, to improve the drug approval process and better serve the patient population suffering from these common forms of cancer.

An imaging technique which has shown promise in treatment response assessment in osteoblastic metastases is diffusion-weighted MRI (DWI), which is based on the Brownian motion of water and can be quantified by the apparent diffusion coefficient (ADC). The ADC is inversely correlated with cellularity [13]. The treatment response of osteoblastic metastases, resulting in the loss of cell membrane integrity and cellular density, has been shown to result in an increase in the ADC in preclinical mouse models, suggesting its potential clinical utility [14,15,16]. The promising results obtained in preclinical models could be replicated by first studies on humans [15,17,18,19,20,21], albeit not by all [22].

It is crucial to be aware that ADC measurements are subject to random measurement variability, as with all quantitative biomarkers. A change in ADC value in longitudinal studies may not necessarily indicate a real change but rather be a result of this random variability. Therefore, before using a quantitative biomarker such as ADC in longitudinal studies, it is essential to assess its measurement repeatability through test–retest studies. Measurement repeatability can be quantified by the repeatability coefficient (RC) or the within-subject coefficient of variation (wCV), which are used in longitudinal studies to determine if a measured difference represents real change or is within the range of expected random measurement variability.

Furthermore, it is important to consider the degree of repeatability in relation to the changes that occur with treatment response or progression. A quantitative marker like the ADC has diagnostic potential only when the differences between the measurements of viable and nonviable metastases are significantly greater than the measurement uncertainty.

The range of ADC values that can be expected in the spectrum from highly viable to nonviable metastases is difficult to determine, since conventional morphologic imaging cannot determine viability of metastases and routine biopsies are not indicated. For our study, we intend to use longitudinal PSMA-PET as a surrogate for viability of bone metastases. PSMA-PET uses radiotracers specifically targeting the prostate-specific membrane antigen (PSMA), a surface protein highly overexpressed by most prostate cancer cells, allowing for highly sensitive and specific detection of prostate cancer manifestations. Recent studies indicate that PSMA-PET is not only highly sensitive in the detection of prostate cancer lesions but that change in PSMA uptake under therapy can serve as a marker for treatment response [23,24,25,26].

So far, there has been only one study investigating the repeatability of ADC measurements of osteoblastic metastases, which has not been corroborated so far. Also, currently, it is unclear what range of ADC values can be expected in viable and nonviable metastases. Therefore, our study aims to further the understanding of ADC measurement repeatability of osteoblastic metastases and to determine how it relates to the range of ADC values seen in viable and nonviable metastases, as determined by PSMA-PET.

## 2. Materials and Methods

### 2.1. Study Design, Patients and Imaging Protocol

Nine men with prostate cancer and known bone metastases, presenting for clinically indicated PSMA-PET between June 2022 and January 2023 at the University Hospital Münster, were included. Patient characteristics are shown in Table 1.

The study was approved by the local ethics committee (2021-825-f-S) and performed in accordance with the 1964 Declaration of Helsinki and its amendments. Written informed consent was obtained from all patients.

First, patients were injected with 3 MBq/kg body weight [^18^F]PSMA-1007 for clinically indicated PSMA-PET. One hour after PET tracer injection, two repeated T1w and DWI MRI measurements were conducted on a Biograph mMR PET-MR-System (Siemens, Erlangen, Germany) to determine in vivo repeatability of DWI measurements. There was no concurrent PET scan at this time. Diffusion acquisition was performed using a Spin-Echo-EPI Sequence (FOV 380 × 275 mm², 21 axial slices, voxel size 2 × 2 × 5 mm³, TE 86 ms, TR 6400 ms, fat suppression SPAIR, 1 × b = 50 s/mm², 3 × 400 s/mm², 3 × 800 s/mm²). ADC-maps were automatically calculated with the standard settings provided by the vendor. For T1, an axial volumetric interpolated breath hold examination (VIBE) sequence was used (FOV: 420 × 342, acquisition matrix: 320 × 224, slice thickness 5 mm, TR 1.96 ms, TR 4.07, no fat suppression). Between the two MRI measurements, the patients were moved out of the MRI, repositioned and moved back in again. The area covered by repeated DWI measurement in each patient can be found in Table 1.

Subsequently, all patients underwent clinically indicated PSMA-PET/CT or PET/MRI scans from skull to tibia two hours after tracer injection. The patients were asked to void their bladder before the PET scan. PET image reconstruction was performed with onboard software using OSEM with 21 subsets and 3 iterations.

Five patients were scanned on the integrated 3-Tesla Biograph PET-MRI, also used for repeated DWI measurements; the other four patients were scanned on a PET/CT System (Biograph mCT, Siemens, Erlangen, Germany). Four of the nine patients had previously undergone PSMA-PET.

### 2.2. Phantom Measurements

MRI-phantom measurements were performed on a commercially available DWI phantom (https://qmri.com, accessed on 12 June 2023) with an MR-readable thermometer to adjust target diffusion values for temperature. Six measurement runs with five repetitions each were performed under identical conditions, using the same MRI sequences and flex body coil as for the patient study. Phantom measurements were evaluated using MIM^®^ Version 7.2.4 (MIM Software Inc., Cleveland, OH, USA).

### 2.3. Image Analysis

To identify bone lesions, b400 DWI images were reviewed for areas of bone marrow hyperintensity relative to background marrow with corresponding hypointensity on T1w [27]. Additionally, MR images were correlated to PSMA-PET. Same-day and previous PSMA-PET served as a surrogate for viability of bone metastases, with metastases showing strong uptake considered viable and those currently showing no uptake but having had strong uptake in pretreatment PET considered nonviable. The osteoblastic nature of metastases was visually confirmed on CT scans of same-day PET/CT in four patients, previous CT in four patients and on radiographs of the spine and pelvis in one patient. Volumes of interest (VOIs) were delineated by a board-certified nuclear medicine specialist on the ADC maps using MINT-Lesion (Mint Medical, Dossenheim, Germany). b50, b400, b800, T1w and PSMA-PET images were used to check for plausibility of the contours. In cases of diffuse disease, the VOI was drawn around the entire vertebral body or pelvic bone [28]. The mean and median ADC values (ADC_mean_ and ADC_median_) within the VOI were measured. The maximum standardized uptake value ($SUV_{max}$) was measured using a VOI on PET images. For lesions showing no tracer accumulation above background, a VOI was drawn in correlation to DWI signal alterations and alterations on T1w images or CT.

### 2.4. Statistical Analysis

Normally distributed data are described using mean ± standard deviation (SD) and non-normally distributed data using median and interquartile range (IQR, 25th–75th percentile). Data were checked for normality using histograms. Association of quantitative variables was analyzed using Spearman’s correlation coefficient ($r_{Spearman}$) or linear regression.

In the phantom measurements, no correlation within the measurement runs was observed. Therefore, all measurements are pooled by concentration. To enable comparability with the in vivo measurements, results are reported as estimates of standard deviation (SD) and repeatability coefficient (RC=2.77×SD), as well as coefficient of variation (CV) [29] and relative repeatability coefficient %RC, defined as 2.77×CV.

For analysis of the repeatability of the in vivo measurements, the lesions are treated as independent subjects, as differences between repeated measurements of lesions showed no relevant correlation within patients. The within-subject standard deviation (wSD) and the resulting repeatability coefficient, as well as the within-subject coefficient of variation (wCV) and the corresponding %RC, are estimated [30,31,32,33].

The agreement of repeated measurements is visualized using the methods of Bland and Altman [34,35]. Agreement is further quantified using an intraclass correlation coefficient (ICC) based on a one-way random effects model [36]. The repeatability of different regions or classes of lesion size is compared using the Kruskal–Wallis test and the Wilcoxon rank-sum test, respectively. The repeatability of ADC_median_ and ADC_mean_ is compared with the Wilcoxon signed-rank test. Two-sided *p*-values are reported for all tests.

In contrast to the differences between repeated measurements, the magnitude of the ADC measurements shows relevant intraindividual correlation within patients. Therefore, the comparison of ADC by PSMA status was performed using a linear mixed model, including a random intercept for both patient and lesion [37]. Since the ADC values were log-transformed for this analysis, the multiplicative factor by which the groups differ regarding their geometric mean is estimated. Model assumptions were assessed using residual plots.

A classical power analysis was not performed. The sample size of 9 patients resulted in 65 lesions that could be included in the analyses. The approach of Danzer et al. [38] allows to determine the effective specificity resulting from a repeatability study as a function of the sample size. The calculation is based on the RC commonly used in the literature for a target specificity of 95% (i.e., RC=2.77×wSD). For 65 lesions that are measured twice, the mean effective specificity is 94.6%. An effective specificity of at least 90% is achieved with a probability of 96.6%.

R version 4.3.0 was used for the analyses [39].

## 3. Results

### 3.1. Phantom Measurements

All phantom measurements were conducted at 22 °C, as indicated by the built-in MR-readable thermometer. For ADC_mean_, the mean deviation from the respective target values over all vials was +$23.6\times 10^{-6}\;{\mathrm{mm}}^{2}/s$ and the mean absolute deviation $27.5\times 10^{-6}\;{\mathrm{mm}}^{2}/s$, with a standard deviation of $17.8\times 10^{-6}\;{\mathrm{mm}}^{2}/s$. The repeatability coefficient averaged over all vials for ADC_mean_ was $54.3\times 10^{-6}\;{\mathrm{mm}}^{2}/s$ and the %RC was 5.83%.

For ADC_median_, the mean deviation from the respective target values over all vials was +$23.9\times 10^{-6}\;{\mathrm{mm}}^{2}/s$ and the mean absolute deviation $27.8\times 10^{-6}\;{\mathrm{mm}}^{2}/s$, with a standard deviation of $17.8\times 10^{-6}\;{\mathrm{mm}}^{2}/s$. The repeatability coefficient over all vials for ADC_median_ was $54\times 10^{-6}\;{\mathrm{mm}}^{2}/s$ and the %RC was 5.81%.

**Figure 1:**
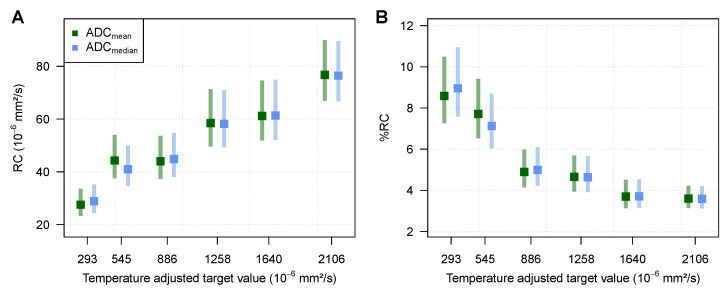
(**A**) Repeatability coefficient (RC) with corresponding 95% confidence intervals of ADC measurements as measured with the phantom (*p*-value of regression slope for target value p=0.001 and p=0.0003 for $ADC_{mean}$ and $ADC_{median}$, respectively). (**B**) %RC with corresponding 95% confidence intervals of ADC measurements as measured with the phantom (*p*-value of regression slope for target value p=0.01 and p=0.01 for $ADC_{mean}$ and $ADC_{median}$, respectively).

As shown in Figure 1, a positive correlation between the ADC target value and the RC was found (*p*-value of regression slope for target value p=0.001 and p=0.0003 for ADC_mean_ and ADC_median_, respectively).

It has been suggested in the literature that the coefficient of variation (CV) and the deducted %RC should be used in the case of a positive correlation between the magnitude of the measurement and the measurement error [33]. Unlike RC, which uses absolute values, %RC expresses the repeatability in proportion to the magnitude of the measurement. However, using %RC in the case of the phantom measurements overcompensated for the positive correlation found between the ADC target value and RC, resulting in a negative correlation between the ADC target value and %RC (Figure 1, *p*-value of regression slope for target value p=0.01 and p=0.01 for $ADC_{mean}$ and $ADC_{median}$, respectively).

Measurement deviations from the respective ADC target values and further results on repeatability stratified by ADC target value can be found in Table A1, Table A2, and Table A3.

### 3.2. Bone Metastases Characteristics

Overall, 87 bone metastases were identified in nine patients. Of those, 14 could be identified in PSMA-PET but not unequivocally on DWI images or ADC maps. Four metastases that could be identified on both PSMA-PET and DWI images had to be excluded from ADC measurements due to movement artifacts. Another 4 metastases had to be excluded because the metastatic nature could not be proven, leaving 65 metastases suitable for ADC measurements.

**Figure 2:**
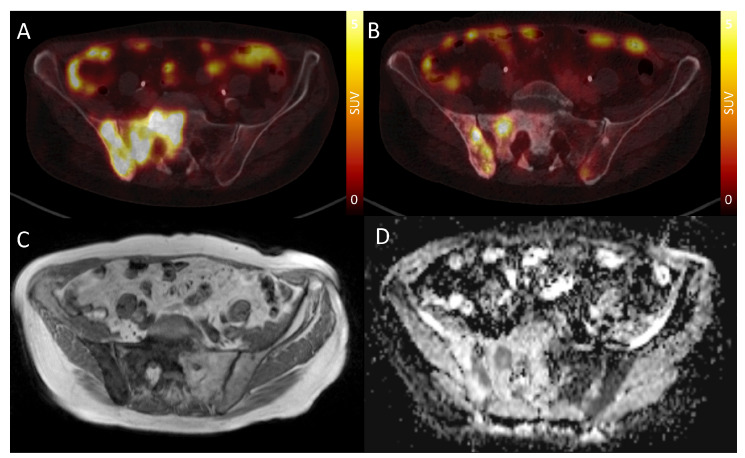
(**A**) PSMA-PET CT of the pelvis in a patient with widespread bone metastases of prostate cancer prior to initiation of PSMA therapy showing homogeneous PSMA uptake of the right iliac and sacral bone. (**B**) The same patient one year later after three cycles of PSMA therapy. Only focal PSMA uptake in the sacral bone and multifocal uptake in the right iliac bone. Large areas of sclerotic bone with perviously high PSMA uptake, now showing at most faint uptake, i.e., considered minimal/nonviable. (**C**) T1w MRI acquired immediately before PSMA-PET in B, showing widespread sclerosis in the right iliac and scaral bone. Note that it is not possible to differentiate areas with high and low uptake in PSMA-PET. (**D**) ADC map of the same location. Note the excellent correlation with PSMA-PET. Areas showing vivid uptake in PSMA-PET are depicted in dark gray, corresponding to ADC values around 1000. Areas with no or minimal uptake are depicted in light gray, corresponding to ADC values from 1300 to 1500.

Six metastases that could be clearly identified on DWI showed only faint uptake on PSMA-PET. Nine metastases could be identified on DWI but showed no uptake on PSMA-PET above background. Of the metastases without discernible tracer accumulation, all showed uptake on previous PSMA-PET examinations (Figure 2). There were 9 metastases in the thoracic spine, 15 in the lumbar spine and 41 in the pelvic region. The detailed location of single metastases can be found in Table A4. The median short axis of the evaluable metastases was 17 mm [IQR: 12–23], and the median long axis was 32 mm [IQR: 19–52]. The median volume of the metastases, as measured on the ADC maps, was 3.1 cm^3^ [IQR: 1.1–12.8]. The median $SUV_{max}$ was 12.1 [IQR: 6.6–17.7].

### 3.3. Repeatability of ADC Measurements in Bone Metastases

The within-subject standard deviation (wSD) for ADC_mean_ and ADC_median_ was $60\times 10^{-6}{\mathrm{mm}}^{2}/s$ and $51\times 10^{-6}\;{\mathrm{mm}}^{2}/s$, respectively (Table 2). Consequently, the repeatability coefficients (RC) for ADC_mean_ and ADC_median_ were determined to be $166\times 10^{-6}\;{\mathrm{mm}}^{2}/s$ and $141\times 10^{-6}\;{\mathrm{mm}}^{2}/s$, respectively. Furthermore, the within-subject coefficient of variation (wCV) for ADC_mean_ and ADC_median_ was 5.5% and 4.7%, respectively.

With the lower wSD and wCV, the repeatability of the in vivo measurements was better for ADC_median_ compared with ADC_mean_ (*p* = 0.04 for comparison of the SD of repeated measurements of ADC_median_ and ADC_mean_). For this reason, the following analyses were conducted using the ADC_median_.

**Figure 3:**
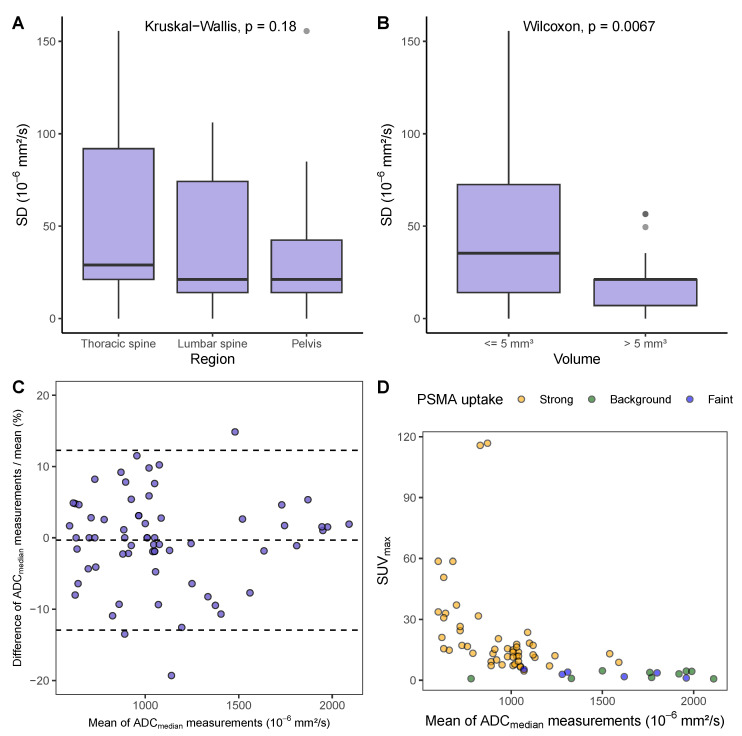
(**A**) Standard deviation (SD) of repeated ADC_median_ measurements by region. (**B**) SD of repeated ADC_median_ measurements dependent on lesion volume. (**C**) Bland–Altman plot of repeated ADC_median_ measurements. The mean of the repeated measurements is plotted against the differences as a percentage of their mean. The distance of the limits of agreement to the mean difference of −0.32% is ±12.60% (95% CI [9.86–15.33]). (**D**) Correlation of average ADC_median_ measurement and SUV_max_, $r_{Spearman}=-0.72$, 95% CI [−0.82–−0.58]).

**Figure A1:**
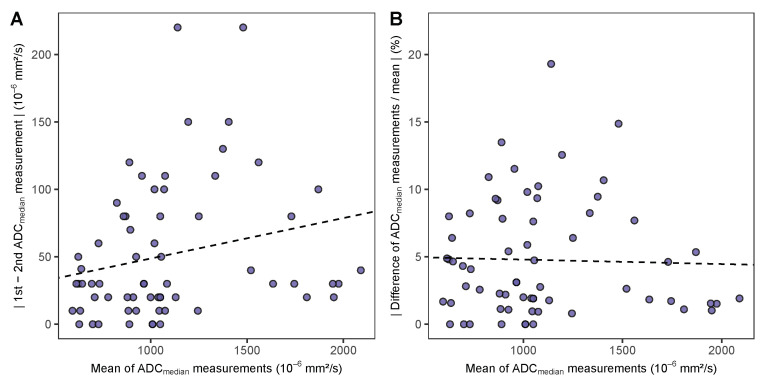
(**A**) Mean of repeated ADC_median_ measurements versus absolute difference of repeated ADC_median_ measurements. The dashed line indicates the linear relationship between mean ADC_median_ and absolute deviation of the repeated measurements. Monotonic association between mean and absolute difference: $r_{Spearman}=0.31,p=0.01$. (**B**) Mean of repeated ADC_median_ measurements versus absolute difference of repeated ADC_median_ measurements as a percentage of their mean. The dashed line indicates the linear relationship between mean ADC_median_ and absolute deviation of the repeated measurements. Monotonic association between mean and absolute difference: $r_{Spearman}=0.008,$p=0.95.

The ICC of 0.983 (95% CI [0.972–0.990]) indicated an excellent agreement of the two ADC_median_ measurements. In the Bland–Altman analysis, the mean difference between the two repeated measurements was $3.09\times 10^{-6}\;{\mathrm{mm}}^{2}/s$, with limits of agreement of $\pm 141.52\times 10^{-6}\;{\mathrm{mm}}^{2}/s$. The Bland-Altman analysis showed that the difference between repeated measurements increased with the height of the measured value (correlation between mean and absolute difference $r_{Spearman}=0.31,p=0.01$, Figure A1). Expressing the differences as a percentage of their average [35] removed the relationship between repeatability and size of the measurement ($r_{Spearman}=0.008,p=0.95$, Figure A1). The distance of the limits of agreement to the mean difference of −0.32% was ±12.60% (95% CI [9.86–15.33], shown in Figure 3), which is very close to the %RC reported in Table 2. Based on these results, wCV and %RC seem suitable to quantify the repeatability of the in vivo measurements.

To investigate whether the anatomical region had an impact on the repeatability of ADC measurements, we compared the standard deviations of repeated measurements in the thoracic spine, lumbar spine and pelvic region. Among the anatomical regions investigated, the standard deviation of repeated ADC_median_ measurements was the highest in the thoracic spine, followed by the lumbar spine, and it was the lowest in the pelvic region (Figure 3). However, the differences were not statistically significant (p=0.18) with corresponding RCs of 209.0, 149.7, and $116.3\times 10^{-6}\;{\mathrm{mm}}^{2}/s$.

To investigate whether metastasis size had an impact on ADC measurement repeatability, we categorized the metastases into those with a volume of less or equal $5\;{\mathrm{mm}}^{3}$ (n = 40) and those with a volume of more than $5\;{\mathrm{mm}}^{3}$ (n = 25). Our analysis showed a difference (p=0.007) in the standard deviation of repeated measurements in the dependence of lesion volume (Figure 3). The RC for lesions $\leq 5\;{\mathrm{mm}}^{3}$ was $170.4\times 10^{-6}\;{\mathrm{mm}}^{2}/s$, and the RC for lesions $>5\;{\mathrm{mm}}^{3}$ was $69.9\times 10^{-6}\;{\mathrm{mm}}^{2}/s$.

### 3.4. ADC Range in Bone Metastases and Association with PSMA-PET Uptake

**Figure 4:**
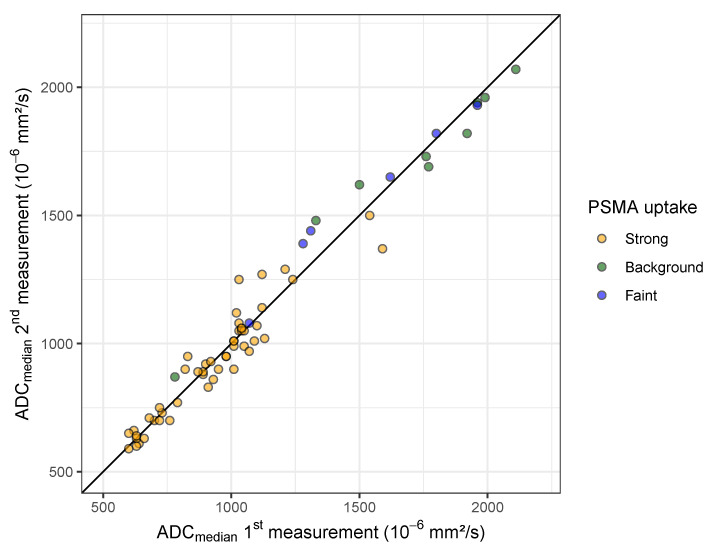
Agreement of ADC_median_ measurements and association with visually assessed uptake of corresponding metastases in PSMA-PET. The diagonal line indicates perfect agreement of the 1st and 2nd measurement. ICC 0.983 (95% CI [0.972–0.990]). Note the small variation between repeated measurements compared with the range of values and the difference between metastases with strong PSMA uptake and those with low tracer uptake. Compared with lesions with strong PSMA uptake, the ADC_median_ is on average 64.1% (95% CI [41.6–90.4], p<0.001) and 63.2% (95% CI [44.6–84.8], p<0.001) higher in lesions with faint tracer uptake and PET signal on background level, respectively, according to a linear mixed-model analysis.

The mean value of repeated ADC_median_ measurements ranged from $595\times 10^{-6}\;{\mathrm{mm}}^{2}/s$ to $2090\times 10^{-6}\;{\mathrm{mm}}^{2}/s$ (Figure 4). The lowest mean ADC_median_ was observed in lesions with strong PSMA uptake with $930\times 10^{-6}\;{\mathrm{mm}}^{2}/s$. A markedly higher mean ADC_median_ was found in lesions with only faint tracer uptake ($1529\times 10^{-6}\;{\mathrm{mm}}^{2}/s$) and with PET signal on background level ($1683\times 10^{-6}\;{\mathrm{mm}}^{2}/s$). According to the linear mixed-model analysis, the ADC_median_ was on average 64.1% (95% CI [41.6–90.4], p<0.001) and 63.2% (95% CI [44.6–84.8], p<0.001) higher in lesions with faint tracer uptake and PET signal on background level, respectively.

The median $SUV_{max}$ of the metastases was 12.1 (IQR: 6.6–17.7). A marked decrease in $SUV_{max}$ with an increase in ADC_median_ can be seen (Figure 3, $r_{Spearman}=-0.72$, 95% CI [−0.82–−0.58]).

## 4. Discussion

A crucial requirement for a quantitative treatment response biomarker is low variability between repeated measures, indicated by a small RC or %RC, relative to the differences observed between pathological states and treatment response. This study assessed the repeatability of ADC measurements in osteoblastic bone metastases compared with the ADC values obtained from viable and nonviable metastases using phantom and in vivo studies. The RC and %RC of ADC measurements were found to be small when compared with the difference observed between viable and nonviable metastases. Therefore, ADC measurements meet the essential technical prerequisite for reliable treatment response evaluation in osteoblastic metastases.

Measurement repeatability of quantitative imaging biomarkers can be quantified using different metrics. It has been suggested that the within-subject standard deviation (wSD) and its derived repeatability coefficient (RC) should be utilized when repeatability is independent of biomarker magnitude. Conversely, for biomarkers where measurement variability increases with larger values, the within-subject coefficient of variation (wCV) and the relative repeatability coefficient (%RC) have been proposed as measures of repeatability [33]. To our knowledge, it has not been investigated so far whether the repeatability of ADC measurements depends on the magnitude of the measured values. We could demonstrate in our phantom and in vivo study that the measurement error does indeed increase with the magnitude of the target value. Using measures of relative change to quantify repeatability allowed to handle this association for in vivo measures and resulted in stable limits over the range of observed values (Figure 3).

Also, the quantification of ADC in a region or volume of interest can be performed using different measures. In the in vivo study, we observed a better repeatability for ADC_median_ compared with ADC_mean_ (p=0.04). In contrast, both measures were equally precise in the phantom study. The absence of a difference in the phantom study can be easily explained. Since the vials in the phantom contain a homogenous fluid, every voxel should have the same ADC value under ideal conditions. Image noise and other artifacts should add a random, symmetrically distributed measurement error. Hence, mathematically, ADC_mean_ and ADC_median_ should be identical in the phantom study under ideal conditions. In contrast, the situation is fundamentally different in vivo. Focal bone lesions are surrounded by fatty bone marrow in older individuals, who represent the majority of patients with osteoblastic metastases. Due to the fat suppression techniques used in diffusion-weighted MRI, fatty regions have a very low ADC, often zero. Hence, inclusion of surrounding fatty bone marrow into the volume of interest, be it due to imperfect segmentation or partial volume effect, will result in significant outliers in the array of ADC values obtained for the measurement. We believe that the better measurement repeatability observed for ADC_median_ in vivo is explained by the robustness of the median, as a measure of the average, to outliers, unlike the arithmetic mean.

To date, only a limited number of studies have explored the repeatability of ADC measurements in bone, particularly in focal lesions exhibiting osteoblastic characteristics. The only other study that we are aware of exploring the repeatability of ADC measurements in osteoblastic metastases is by Reischauer et al., investigating 34 pelvic bone metastases of prostate cancer in twelve men. Employing a monoexponential fitting for ADC calculation, a wCV of 4.4% was reported for ADC_median_, closely aligning with the 4.7% found in our study [40]. Messiou et al. investigated the repeatability of ADC measurements in the pelvic bone marrow of nine healthy volunteers, with a repeated scan performed within a 7-day interval. Their estimated %RC of 14.8% is well in line with our results. Their Bland–Altman limits of agreement of mean ADC of bone marrow in normal subjects, however, are much narrower for absolute measurements ($2.0\pm 86\times 10^{-6}\;{\mathrm{mm}}^{2}/s$), possibly due to a much smaller range of measured values [28]. Donners et al. assessed the value of multiparametric MRI to identify viable bone metastases for biopsies. In their sample of 43 lesions, they observed lower, though not statistically significant, mean/median ADC in tumor-positive biopsies [41]. In contrast to the studies by Reischauer et al. [40], Messiou et al. [28] and Donners et al. [41], which employed regions of interest (ROI), we used volume of interests (VOIs). Going beyond previous studies, the validity of ADC measurements were ensured in our study by the use of a phantom containing the complete range of measured values found in vivo.

As previously emphasized, it is crucial not only to ascertain the absolute values of repeatability but also to assess the relative magnitude of repeatability in comparison with the differences observed in measurements between pathological states and treatment responses. In our study, we used PSMA-PET as a surrogate marker of bone lesion viability. Lesions with strong, focal tracer accumulation above background were considered viable. Lesions that were detectable on DWI-MRI but did not show tracer uptake on concurrent PSMA-PET, but had shown focal tracer accumulation on previous PSMA-PET prior to cancer treatment, were considered nonviable, i.e., showing treatment response. In our study, the lesions with focal tracer accumulation had a mean ADC_median_ of $930\times 10^{-6}\;{\mathrm{mm}}^{2}/s$, and those with uptake on background level one of $1683\times 10^{-6}\;{\mathrm{mm}}^{2}/s$. In relative terms, the ADC_median_ of metastases considered nonviable was 63% higher than in those considered viable. Accordingly, a negative correlation between the ADC_median_ and SUV_max_ of bone lesions could be shown. Our results corroborate the findings of Perez-Lopez et al., correlating ADC values to the detectability of cancer cells in 43 histologic samples of osteoblastic bone metastases. Median ADC was significantly lower in biopsies with tumor cells versus nondetectable tumor cells ($898\times 10^{-6}\;{\mathrm{mm}}^{2}/s$ vs. $1617\times 10^{-6}\;{\mathrm{mm}}^{2}/s,p<0.001)$. Tumor cellularity was inversely correlated with ADC (*p* < 0.001). In serial biopsies taken in three patients before and after treatment, changes in MRI parameters paralleled histological changes [13]. The same group also showed a correlation between change in median ADC of bone metastases and treatment response in metastasized prostate cancer [21].

We found a moderate inverse correlation between the ADC and tracer uptake of bone metastases in PSMA-PET, quantified by SUV_max_. To our knowledge, this is the first study investigating this relationship. The imperfect correlation indicates that DWI and PSMA-PET could have a complementary value in treatment response assessment in prostate cancer metastases. Further studies should investigate this possibility. Going beyond PSMA-PET, ADC quantification might allow for treatment response assessment in osteoblastic metastases of breast cancer and other cancers which do not express PSMA.

Our study has limitations. For one, there were only a limited number number of metastases in the thoracic spine. Also, we used PSMA-PET uptake as a surrogate for viability, which is not a perfect gold standard. Still, our results are in very close alignment to those of Perez-Lopez et al., who had histology available [13]. Moreover, interscanner variability was not examined in our study. However, our study establishes a groundwork for future investigations into interscanner variability, i.e., reproducibility. A comprehensive analysis of repeatability serves as a fundamental step in assessing reproducibility, since repeatability limits the extent of agreement achievable when comparing different scanners [35].

## 5. Conclusions

In conclusion, ADC measurements demonstrate a favorable repeatability in relation to the differences found between viable and nonviable metastases. This fulfillment of essential metrological prerequisites establishes a reliable foundation for assessing treatment response in osteoblastic metastases.

## Figures and Tables

**Table 1:** Patient characteristics.

ID	Age	PSA ^1^	Pretreatments									Area Covered by DWI		
	Years	ng/mL	Px	RTx	LHRH	Abi	Enza	PSMA	RTxB	CTX	Apa	Thorax	Lumbar	Pelvis
1	77	0.34	x	x	x	x	x	x	x	x		x	x	x
2	79	110.00	x	x	x	x	x	x	x	x			x	x
3	83	323.00			x	x	x					x	x	x
4	73	3.87		x	x	x			x	x				x
5 ^*^	53	3.98			x	x				x			x	x
6	65	110.00	x	x	x	x			x	x				x
7	78	407.00	x	x	x	x	x			x			x	x
8	67	3.02	x	x	x						x		x	
9	74	1.49	x	x										x

^1^ measured at time of scan. PSA = prostate-specific antigen, Px = prostatectomy, RTx = radiation therapy to prostate region, LHRH = treatment with LHRH agonists, Abi = abiraterone, Enza = enzalutamide, PSMA = 177Lu-PSMA therapy, RTxB = radiation therapy of bone metastases, CTX = taxane-based chemotherapy, Apa = apalutamide, DWI = diffusion-weighted imaging. * Patient five was also treated with Denusomab.

**Table 2:** Repeatability of ADC Measurements in Bone Metastases.

	ADC_mean_	ADC_median_
wSD ($10^{-6}\;{\mathrm{mm}}^{2}/s$)	59.95 (51.18–72.37)	50.71 (43.29–61.22)
RC ($10^{-6}\;{\mathrm{mm}}^{2}/s$)	166.18 (141.87–200.61)	140.56 (120.00–169.68)
wCV (%)	5.51 (4.57–6.69)	4.65 (3.85–5.66)
%RC (%)	15.27 (12.66–18.55)	12.90 (10.68–15.70)

wSD: within-subject standard deviation, RC: repeatability coefficient, wCV: within-subject coefficient of variation. Numbers in brackets are 95% confidence intervals.

## Data Availability

The data are not publicly available because the informed consent signed by the patients did not provide for it.

## Abbreviations

The following abbreviations are used in this manuscript:

ADC	apparent diffusion coefficient
CI	confidence interval
CT	computed tomography
CV	coefficient of variation
DWI	diffusion-weighted (magnetic resonance) imaging
ICC	intraclass correlation coefficient
IQR	interquartile range
MRI	magnetic resonance imaging
PET	positron emission tomography
PSA	prostate-specific antigen
PSMA	prostate-specific membrane antigen
PVP	polyvinylpyrrolidone
RC	repeatability coefficient
RECIST	Response Evaluation Criteria in Solid Tumors
SD	standard deviation
SUV	standardized uptake value
VOI	volume of interest
wCV	within-subject coefficient of variation
wSD	within-subject standard deviation

## Appendix A

**Table A1:** Deviation of ADC_mean_ and ADC_median_ measurements from the target value in the phantom stratified by ADC target values.

		ADC_mean_		ADC_median_	
PVP Concentration	Temperature-Adjusted Target Value	Mean Deviation from Target Value	Mean Absolute Deviation from Target Value	Mean Deviation from Target Value	Mean Absolute Deviation from Target Value
%	$10^{-6}\;{\mathrm{mm}}^{2}/s$	$10^{-6}\;{\mathrm{mm}}^{2}/s$	$10^{-6}\;{\mathrm{mm}}^{2}/s$	$10^{-6}\;{\mathrm{mm}}^{2}/s$	$10^{-6}\;{\mathrm{mm}}^{2}/s$
0	2106.00	33.81	36.28 (24.33)	33.58	36.07 (24.20)
10	1640.00	21.69	25.20 (17.88)	21.90	25.50 (17.78)
20	1258.00	3.50	18.54 (10.38)	3.45	18.38 (10.44)
30	886.00	16.80	19.09 (12.97)	17.08	19.52 (13.09)
40	545.00	31.46	32.17 (14.45)	32.38	32.68 (14.09)
50	293.00	29.13	29.13 (9.93)	30.27	30.27 (10.40)

All measurements were conducted at a temperature of 22 °C, as indicated by the built-in MR-readable thermometer. PVP: polyvinylpyrrolidone. Numbers in brackets are standard deviations.

**Table A2:** Deviation and repeatability of ADC_median_ measurements in the phantom, stratified by ADC target values.

PVP Concentration	Temperature-Adjusted Target Value	Number of Measurements	SD	RC	CV	%RC
%	$10^{-6}\;{\mathrm{mm}}^{2}/s$		$10^{-6}\;{\mathrm{mm}}^{2}/s$	$10^{-6}\;{\mathrm{mm}}^{2}/s$	%	%
0	2106.00	6 × 5 × 3	27.6 (24.1–32.3)	76.5 (66.7–89.6)	1.3 (1.1–1.5)	3.6 (3.1–4.2)
10	1640.00	6 × 5 × 2	22.1 (18.8–27.0)	61.4 (52.0–74.8)	1.3 (1.1–1.6)	3.7 (3.1–4.5)
20	1258.00	6 × 5 × 2	21.0 (17.8–25.6)	58.2 (49.3–71.0)	1.7 (1.4–2.0)	4.6 (3.9–5.7)
30	886.00	6 × 5 × 2	16.2 (13.7–19.7)	44.9 (38.0–54.7)	1.8 (1.5–2.2)	5.0 (4.2–6.1)
40	545.00	6 × 5 × 2	14.8 (12.5–18.0)	41.0 (34.7–50.0)	2.6 (2.2–3.1)	7.1 (6.0–8.7)
50	293.00	6 × 5 × 2	10.4 (8.8–12.7)	28.8 (24.4–35.2)	3.23 (2.7–4.0)	9.0 (7.6–11.0)

All measurements were conducted at a temperature of 22 °C, as indicated by the built-in MR-readable thermometer. PVP: polyvinylpyrrolidone, SD: standard deviation, RC: repeatability coefficient, CV: coefficient of variation. Number in brackets are 95% confidence intervals.

**Table A3:** Deviation and repeatability of ADC_mean_ measurements in the phantom, stratified by ADC target values.

PVP Concentration	Temperature-Adjusted Target Value	Number of Measurements	SD	RC	CV	%RC
%	$10^{-6}\;{\mathrm{mm}}^{2}/s$		$10^{-6}\;{\mathrm{mm}}^{2}/s$	$10^{-6}\;{\mathrm{mm}}^{2}/s$	%	%
0	2106.00	6 × 5 × 3	27.7 (24.2–32.5)	76.8 (66.9–90.0)	1.3 (1.1–1.5)	3.6 (3.1–4.2)
10	1640.00	6 × 5 × 2	22.1 (18.7–26.9)	61.1 (51.9–74.6)	1.3 (1.1–1.6)	3.7 (3.1–4.5)
20	1258.00	6 × 5 × 2	21.1 (17.9–25.7)	58.5 (49.6–71.3)	1.7 (1.4–2.1)	4.7 (3.9–5.7)
30	886.00	6 × 5 × 2	15.9 (13.5–19.4)	44.0 (37.3–53.7)	1.8 (1.5–2.2)	4.9 (4.1–6.0)
40	545.00	6 × 5 × 2	16.0 (13.5–19.5)	44.3 (37.5–54.0)	2.8 (2.4–3.4)	7.7 (6.5–9.4)
50	293.00	6 × 5 × 2	9.9 (8.4–12.2)	27.5 (23.3–33.6)	3.1 (2.6–3.8)	8.6 (7.3–10.5)

All measurements were conducted at a temperature of 22 °C, as indicated by the built-in MR-readable thermometer. PVP: polyvinylpyrrolidone, SD: standard deviation, RC: repeatability coefficient, CV: coefficient of variation. Number in brackets are 95% confidence intervals.

**Table A4:** Detailed location of metastases measured.

**Thoracic Spine**											
**(n = 9)**											
T1 0	T2 0	T3 0	T4 2	T5 1	T6 1	T7 1	T8 1	T9 0	T10 1	T11 1	T12 1
**Lumbar Spine**											
**(n = 15)**											
L1 3		L2 2			L3 6		L4 2			L5 2	
**Pelvis**											
**(n = 41)**											
right iliac bone 13	left iliac bone 7		sacral bone 12	right pubic bone 1	left pubic bone 2		right ischium 2	left ischium 2	right femur 1		left femur 1
